# Neurosensory and Cognitive Modifications in Europe's Toughest RandoRaid Competition: the Transpyrénéa Extreme Study

**DOI:** 10.3389/fphys.2017.00201

**Published:** 2017-04-04

**Authors:** Alessandro Tonacci, Simona Mrakic-Sposta, Kristian Ujka, Francesco Sansone, Alice Ferrisi, Guido Giardini, Raffaele Conte, Lorenza Pratali

**Affiliations:** ^1^Institute of Clinical Physiology, National Research Council (IFC-CNR)Pisa, Italy; ^2^Institute of Bioimaging and Molecular Physiology, National Research CouncilSegrate, Italy; ^3^Department of Psychology, University of TorinoTorino, Italy; ^4^Mountain Medicine Center, Valle d'Aosta Regional Hospital Umberto PariniAosta, Italy

**Keywords:** cognition, extreme physiology, neurosensory assessment, olfaction, smell

## Abstract

**Introduction:** Given the wide proliferation of ultra-long endurance races, it is important to understand the physiological response of the athletes to improve their safety. We evaluated the cognitive and neurosensory effects on ultra-endurance athletes during the Transpyrénéa (866 Km, 65,000 m positive slope), held on the French Pyrenees.

**Materials and Methods:** 40 athletes were enrolled (age 43.8 ± 8.8 years; 36 males). Olfactory and cognitive tests were performed before the race (T0, *n* = 40), at 166 kms (T1, *n* = 28), at 418 kms (T2, *n* = 20), and after the race (T3, 866 kms, *n* = 13). The effect of dehydration and sleep deprivation on cognitive features were also studied.

**Results:** Olfactory function decreased during the race (T0: 24.9 ± 4.3 vs. T3: 22.8 ± 3.5, *z* = -2.678, *p* = 0.007), language fluency increased (T0: 10.8 ± 2.9; T1: 11.4 ± 2.7; T2: 12.9 ± 2.8; T3: 12.9 ± 3.0; χ^2^ = 11.132, *p* = 0.011 for combined samples), whereas the Trail Making Test did not show any changes between pre- and post-race (T0 vs. T3 *p* = 0.697 for TMT-A, *p* = 0.977 for TMT-B). The mean aggregate sleeping time was 9.3 ± 5.4 h at T1, 22.4 ± 10.0 h at T2, 29.5 ± 20.5 h at T3, with a correlation with olfactory function (*r* = 0.644, *p* = 0.018), while Total Body Water (TBW) was not correlated with olfactory or cognitive scores.

**Conclusion:** Physical activity and sleep restriction in ultra-endurance could transiently affect olfactory function, while verbal fluency improved, demonstrating a dissimilar mechanism of activation/deactivation in different cortical areas. Body water loss was uncorrelated to cognition. Further studies should clarify whether cognitive and sensory deficits occur even in absence of sleep restriction.

## Introduction

RandoRaids are competitions that can be considered a mix between the so-called “Chemin Solidaires,” the “Grandes Randonnées” and the Non-Stop Raid Nature, often conducted in semi-self-sufficiency and run for extremely long distances. Differently from ultra-trail marathons, RandoRaids are largely conducted in independence by the raiders, and cover longer distances.

Often completed in times that are somewhat similar to the ultra-trail marathon [the minimum average speed to complete the Tor des Géants ultra-trail (330 Km) is 2.2 km/h, while this value is set to 2.165 km/h for the Transpyrénéa RandoRaid (866 Km)], it is reasonable to think that effects on the health status of competitors would be similar to those experienced by the ultra-trailers. To date, various evidence for significant effects on physical (Vitiello et al., 2013) and cognitive functions (Tomporowski, 2002; Cona et al., 2015; Hurdiel et al., 2015; Tonacci et al., 2016) of ultra-trailers has already been observed, mainly caused by a mix of high altitude exposure (Yan, 2014), environmental conditions (Lefferts et al., 2016; Taylor et al., 2016)—including cold, heat and hypoxia—muscular fatigue, dehydration (Cian et al., 2000), and sleep deprivation (Davis et al., 2014; Fullagar et al., 2015).

According to Hurdiel et al. (2015), ultra-trailers experience a wide range of symptoms, ranging from response lags to cognitive tasks to serious symptoms such as visual hallucinations, regardless of rest duration, and time in race. In addition, neurosensory features appear to be affected by a mix of strenuous physical effort and sleep restriction/deprivation, as demonstrated in a recent study held during the Tor des Géants ultra-trail (Tonacci et al., 2016).

Cognitive reserve also turns out to be linked to physical performances, as demonstrated by Cona et al. (2015), which found the correlation between ultra-trailers' physical performances and their neurocognitive functioning, including superior inhibitory control for motor response and for processing of irrelevant information (i.e., emotional stimuli). Such findings are somehow in line with results achieved by Del Percio et al. (2009), which hypothesized a mechanism of neural efficiency ruling the performances of elite athletes also in different sport activities.

However, the cognitive abilities of athletes performing ultra-long distances—and RandoRaids in particular—are not well-explored in scientific literature, and thus present an interesting challenge to be investigated by studies conducted in naturalistic settings.

In particular, focusing on cognitive and neurosensory features, such domains are normally studied via exhaustive tests that take time to administer, so are unsuitable for longitudinal administration during the execution of a competition. However, thanks to surrogate biomarkers and quick-to-administer testing methods, it is possible to retrieve interesting information regarding the cognitive and sensory status of an athlete, even during a strenuous competition such as an ultra-long RandoRaid. The aim of our study was to evaluate the cognitive and neurosensory effects on ultra-endurance RandoRaid athletes during the Transpyrénéa race.

## Materials and methods

The Transpyrénéa first took place in the summer of 2016 on the French side of the Pyrenees, covering an overall distance of 866 km with 65,000 meters of positive slope throughout the race. The race started near the Mediterranean Sea, in Le Perthus, and ended in Hendaye, on the Atlantic Ocean, following the Grande Randonnée path called GR10. The maximum time to complete the race was set to 400 h, with 22 checkpoints (CPs) and three life bases (LBs, 166, 418, 678 km) along the path.

### Subjects

A total of 40 athletes (36 males, mean age: 43.8 ± 8.8 years) participated as volunteers in this study (Table 1). Exclusion criteria were the presence of conditions possibly affecting smell (neurodegeneration, history of head trauma, endocrine disorders, flu, and/or nasal problems), use of medications or unwillingness to give informed consent.

**Table 1:** **Study population**.

Number of subjects	40
Age (years, mean ± SD)	43.8 ± 8.8
Male gender, n (%)	36 (90)
Height (cm, mean ± SD)	176.1 ± 7.4
Smoking habit, n current/n former smokers (%)	2/4 (5/10)
Diabetes, n (%)	1 (2.5)
Hypertension, n (%)	3 (7.5)
Training, years (mean ± SD)	19.6 ± 15.5
Weekly training (h/week, mean ± SD)	11.5 ± 6.7

This information was retrieved through a structured questionnaire administered to all participants prior to the race. The questionnaire also included questions on hypertension, hypercholesterolemia, diabetes, and smoking, as well as on physical activity.

The study was approved by the institutional Ethics Committee of the Aosta Valley Hospital (n. 895; 31/8/2015) and followed the guidelines of the Helsinki Declaration. All volunteers were informed regarding the design and purposes of the study and gave written informed consent.

### Experimental overview

Four testing sessions were planned for the study. The first session, “Baseline” (T0), took place in Le Perthus (km 0, altitude 420 m above sea level) 1 or 2 days before the race depending on the availability of the athletes. After the first quarter of the race, a second session was held at the LB in Mérens-les-Vals (T1, km 166, altitude 1,060 m above sea level), while at halfway a third session was managed at the LB in Bagnères-de-Luchon (T2, km 418, altitude 640 m above sea level). At the end of the race, in Hendaye (T3, km 866, altitude 0 m above sea level), athletes were evaluated for the fourth time, within an hour of their arrival and concluding any physical effort. All the subjects enrolled were assessed within an hour of concluding the race.

#### Neurosensory evaluation: olfactory assessment

Olfactory assessment was performed by a simple, reliable odor identification task, Sniffin' Sticks (Burghart Medizintechnik GmbH, Wedel, Germany), already shown to be employable in a naturalistic setting including the ultra-trail marathons (Tonacci et al., 2016).

The test administration was simple: each odor, contained within a felt-tip pen, was placed nearby the nostrils of the volunteer for 3s. The athlete was asked to identify each of the odors presented within a set of four answers each (the correct one and three confounders). In this study, both the “Blue,” traditional version of the test (Haehner et al., 2009), and the “Purple Identification Plus” extension (Sorokowska et al., 2015) were employed, so that the number of odors presented to the athletes was 32, with the test score, ranging from 0 to 32, indicating the overall number of correct answers given.

#### Cognitive assessment: language control and executive function evaluation

Language assessment was performed in all testing sessions via a shortened version of the Controlled Oral Word Association Test (COWAT; Benton and Hamsher, 1989), chosen for its simple and quick administration, extremely compliant with the time requirements of the athletes undergoing this strenuous competition. Specifically, the COWAT is a verbal fluency test considered to be a measure of executive function. It requires participants to say as many words they can think of which begin with a particular letter of the alphabet, administered within a time limit of 30 s.

A computerized version of the Trail Making Test (TMT) was administered at T0 and at race completion (T3). Intermediate sessions were not included for TMT, as they were time-consuming for the race management of athletes. This cognitive test was performed in its entirety, consisting of two parts, namely TMT-A and TMT-B. Specifically, the first part (TMT-A) assesses cognitive processing speed, attention and sequencing abilities, whereas the second part (TMT-B) is mainly related to executive functions (Bowie and Harvey, 2006). TMT-A requested participants to connect circles labeled with numbers 1–25 in ascending order. In TMT-B, participants should alternate between numbers (from 1 to 13) and letters (from A to L) in an ascending order (i.e., 1-A-2-B and so on).

To ensure full comprehension of the test instructions, participants were provided with computer-based instructions and short test trials.

In this study, the total time needed to complete each of the two tests, their sum and the ratio between TMT-B and TMT-A, known to be an accurate measure of executive functions (Hester et al., 2005), were calculated.

#### Body composition analysis

Several indices related to body composition, including body weight, body mass index (BMI) and total body water (TBW), were measured using the bioelectrical impedance analysis (Tanita SC-331S Body Composition Analyzer; Tanita Inc., ArlingtonHeights, IL, USA). The subjects evaluated had no drink or food restrictions during the race.

#### Sleep management assessment

The number of hours of waking/sleep were asked of each participant with a structured questionnaire. A total sleep time (TST) was then calculated, together with a normalized total sleep time (NTST), as the ratio between TST and race duration, this latter parameter extracted from the official timing of the competition.

### Statistical analysis

Statistical analysis was performed using SPSS17 software (SPSS Inc., Chicago, IL, USA). As a first step, we applied the Shapiro-Wilk test to check for the normality of the variables considered. Since the variables had a non-normal distribution, we employed a non-parametrical multivariate repeated measures analysis of variance (Friedman's test). For *post-hoc* analysis, the Wilcoxon signed-rank test for paired samples was carried out to compare olfaction in the different phases of the competition. Spearman's rank test was used for correlations, in particular concerning olfaction, cognitive parameters, sleep, body composition and race variables (race duration, kms walked, and so forth).

## Results

Of the 40 athletes initially enrolled, 28 subjects (25 males; age 43.5 ± 8.5 years) reached Mérens-les-Vals (after an average of 60.0 ± 21.3 h) and were re-evaluated. Twenty of them (17 males, age 44.8 ± 8.7 years) successfully reached Bagnères-de-Luchon after 185.9 ± 23.7 h and were re-assessed, while 13 (11 males; age 43.8 ± 7.6 years) completed the competition in 363.3 ± 33.1 h (Table 2).

**Table 2:** Characteristics of the study population throughout the race (^*^: significant between T0 vs. T1, ^◦^: significant between T0 vs. T3; ^**§**^: significant for combined samples, at ***p*** < 0.05).

**Variable**	**T0**	**T1**	**T2**	**T3**
Number of subjects	40	28	20	13
Age (years, mean ± SD)	43.8 ± 8.8	43.5 ± 8.5	44.8 ± 8.7	43.8 ± 7.6
Male gender, n (%)	36 (90)	25 (89.2)	17 (85)	11 (84.6)
**COMPETITION STATISTICS**				
Hours walked (mean ± SD)	0.0 ± 0.0	60.0 ± 21.3	185.9 ± 23.7	363.3 ± 33.1
**BODY COMPOSITION ANALYSIS**				
Weight (kg, mean ± SD)	72.6 ± 10.9	70.6 ± 9.5	70.3 ± 8.9	68.1 ± 9.8
BMI (kg/m^2^, mean ± SD)	23.3 ± 2.6	22.6 ± 2.4	22.9 ± 1.8	21.3 ± 1.6
Fat mass (kg, mean ± SD)	5.4 ± 3.5	3.7 ± 2.2	2.9 ± 2.6	2.3 ± 0.9
Total body water (kg, mean ± SD)	49.2 ± 7.2	49.0 ± 6.4	49.6 ± 5.7	46.2 ± 7.0
**SLEEP ANALYSIS**				
Total sleep time (hours, mean ± SD)	0.0 ± 0.0	9.3 ± 5.4	22.4 ± 10.0	29.5 ± 20.5
Normalized total sleep time (% of the total race time, mean)	0.0	14.9	13.6	9.6
Olfactory identification score (mean ± SD)	24.9 ± 4.3^*◦^	23.3 ± 3.9^*^	22.6 ± 3.7	22.8 ± 3.5^◦^
Shortened COWAT score (mean ± SD)	10.8 ± 2.9^◦§^	11.4 ± 2.7^§^	12.9 ± 2.8^§^	12.9 ± 3.0^◦§^
TMT-A (ms, mean ± SD)	57120.5 ± 36539.0			54540.1 ± 12294.6
TMT-B (ms, mean ± SD)	62676.2 ± 25749.9			62488.6 ± 19303.6
TMT-B/A (ratio, mean ± SD)	1.199 ± 0.450			1.138 ± 0.183

All the athletes entering the life bases for testing decided to undergo both the olfactory and cognitive assessments. The mean time for the complete test battery administration was 9.5 ± 3.5 min at T0, 7.5 ± 2.3 min at T1 and T2, 9.5 ± 2.4 min at T3.

### Olfactory evaluation

**Figure 1:**
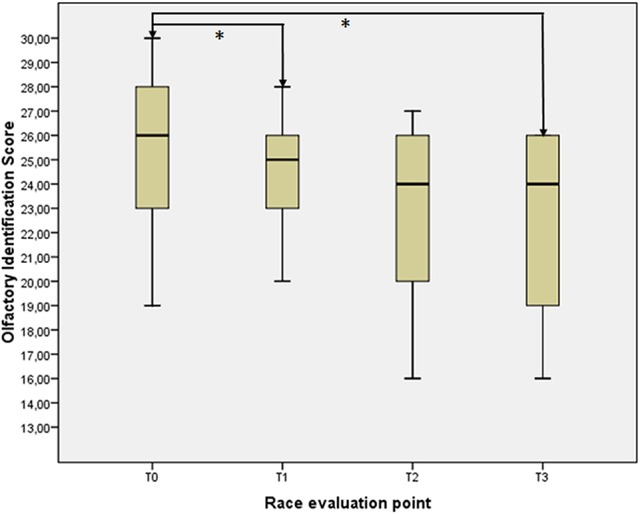
**Olfactory performances throughout the race**.

The olfactory performances throughout the race are displayed in Figure 1. A slight, non-significant trend toward a decrease in odor identification ability was seen during the race (*p* = 0.108, χ^2^ = 6.082 for combined samples). Conversely, the differences between T0 and T1 (*z* = -2.539, *p* = 0.011), as well as between T0 and T3 (*z* = −2.678, *p* = 0.007) were largely significant, whereas between T1 and T2 (*z* = −0.096, *p* = 0.924) and between T2 and T3 (*z* = −0.851, *p* = 0.395) no particular trend was noticed.

Olfactory function was somehow age-dependent (*r* = −0.300, *p* = 0.063 at T0), but independent from gender (possibly due to the low number of female subjects composing the experimental sample) or training habits. Furthermore, olfactory identification ability at T0 was not an early marker of good success in the race, with no significant differences between finishers and non-finishers (*z* = −0.784, *p* = 0.449) in this task.

### Cognitive assessment

**Figure 2:**
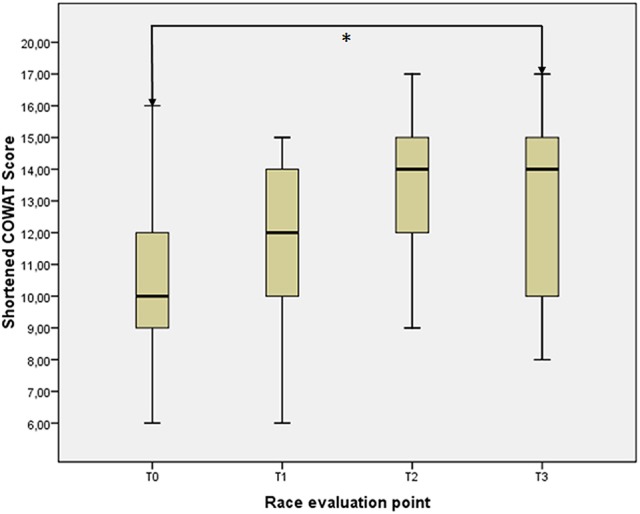
**Shortened COWAT test scores throughout the race**.

The shortened COWAT test for verbal fluency displayed an increase in the overall score throughout the race (*p* = 0.011, χ^2^ = 11.132 for combined samples, Figure 2), with trends near significance between T0 and T1 (*z* = -1.627, *p* = 0.104) and between T1 and T2 (*z* = −1.663, *p* = 0.096) and significance reached when comparing scores at T0 and T3 (*z* = −2.742, *p* = 0.006). On the other hand, no particular difference was seen between T2 and T3 (*z* = −0.212, *p* = 0.832).

Concerning the TMT test, no differences were seen between the two testing sessions (at T0 and T3), with scores at TMT-A (*z* = −0.114, *p* = 0.910), TMT-B (*z* = −0.341, *p* = 0.733) and TMT-B/A ratio (*z* = 0.000, *p* = 1.000) that were superimposable between the two evaluation points.

### Correlation between olfaction and cognition

**Figure 3:**
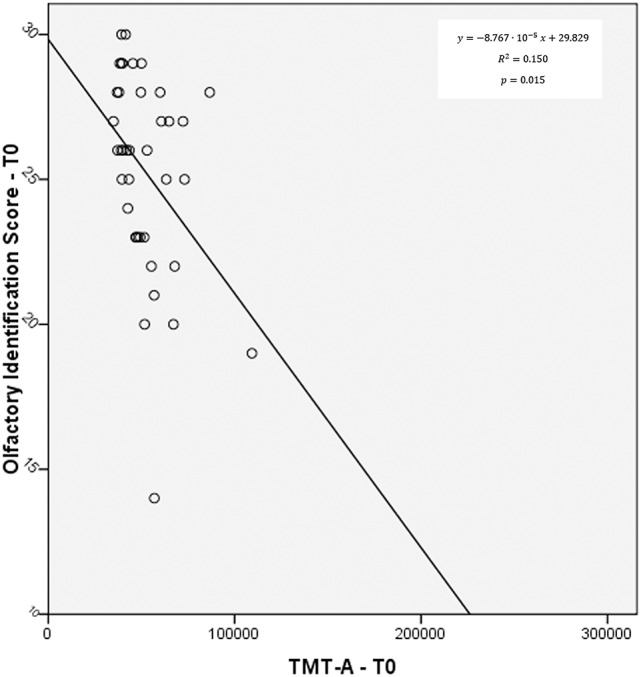
**Correlation between olfactory identification and TMT-A at T0**.

A slight correlation was seen between olfactory identification score at T0 and TMT-A score at the same evaluation point (*r* = −0.388, *p* = 0.015), suggesting a possible link between the two domains studied (Figure 3). No significant correlation was otherwise found between olfactory function and language fluency.

Not very surprisingly, at T3 language fluency and TMT-B/A ratio were largely correlated (*r* = −0.799, *p* = 0.002), unlike what occurred at T0, where only a small, non-significant trend relating the two variables was observed (*r* = −0.241, *p* = 0.164).

### Correlation between olfaction, cognition, and sleep management

**Figure 4:**
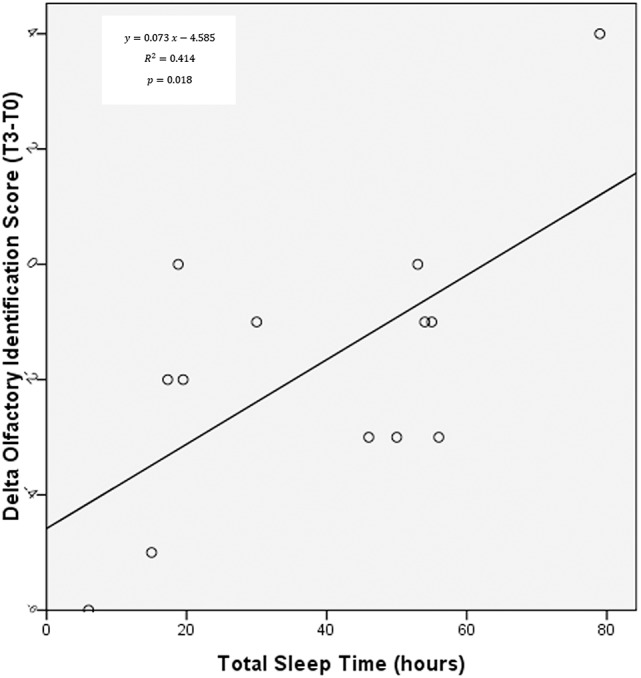
**Correlation between olfactory identification delta (calculated between T3 and T0) and TST**.

A clear, significant Correlation was found between olfactory decrease throughout the race (between T3 and T0) and both TST (*r* = 0.644, *p* = 0.018, Figure 4) and NTST (*r* = 0.639, *p* = 0.025), proving that longer sleep times are more likely to produce smaller decrements in olfactory function during an extreme effort like the Transpyrénéa RandoRaid. No correlations were otherwise seen between TST or NTST and cognitive variables.

### Correlation between olfaction, cognition, and body composition

No significant correlations were found between olfactory or other cognitive scores nor their deltas with body composition variables (body weight, BMI, TBW, and their deltas) at any time.

## Discussion

This study investigated olfactory and cognitive modifications occurring during the first-ever edition of the toughest RandoRaid competition in Europe, Transpyrénéa, organized in the French Pyrenees in summer 2016. The study is a completion of a research previously published about the Tor des Géants® mountain ultra-trail marathon (Borghini et al., 2015; Mrakic-Sposta et al., 2015; Tonacci et al., 2016), adding to the referenced studies a deeper insight into cognition and the use of minimally obtrusive methods, shown to be employable in a naturalistic setting, in a completely different competition. In fact, Transpyrénéa is a much longer competition (866 vs. 330 kms) than the previous one, and therefore requires a dissimilar race management concerning sleep/wake cycles, feeding and so forth. Furthermore, environmental conditions and altimetry profiles are not comparable between the two races, thus representing completely different models to be studied.

Focusing in particular on the cognitive and sensory effects that a similar effort could have on athletes' health, several variables should be taken into account. At first, the effort itself should be considered, since prolonged strenuous physical activity could possibly bring on consequences for health, and specifically cognition and sensoriality, in athletes (Tonacci et al., 2016). Second, mechanisms of sleep deprivation or even sleep restriction are likely to deeply impact, possibly in a transient way, the cognitive and sensory features of a person (Fullagar et al., 2015). Such effects seem to be more dramatic when sleep deprivation is combined with a massive physical effort, as occurs in these events (Davis et al., 2014; Hurdiel et al., 2015), also carrying on serious symptoms, including visual hallucination, possibly impacting the safety of athletes.

Third, feeding and all mechanisms of dehydration and missed/unbalanced supply of nutrients (Murray et al., 2009) that could occur in a similar event could possibly be related to a short-term cognitive deficiency. Fourth, altitude (Yan, 2014), hypoxia, heat and cold stress, known to be both task- and severity-dependent, are probably linked to the cognitive and sensory status of an athlete (Ruffini et al., 2015; Taylor et al., 2016) and are experienced by RandoRaid and ultra-trail athletes competing in a race like the Transpyrénéa.

Therefore, this study employed olfactory testing, a minimally obtrusive, quick, user-friendly surrogate of overall cognitive function and a reliable method for assessing a specific sensory domain, along with specific cognitive testing methods to assess the cognition states of 40 athletes competing in the Transpyrénéa.

Four testing sessions were set up, at the departure (T0), after 166 (T1) and 418 kms (T2) and at the finish line (T3). To avoid particular annoyance for the athletes, a complete testing battery was administered at T0 and T3, whereas at T1 and T2 only olfactory testing and COWAT testing for verbal fluency were performed, with the computer version of TMT performed by athletes only at T0 and T3.

Olfaction—at pre-race assessment slightly related to the age of athletes, as already demonstrated (Kobal et al., 2000)—was seen as slightly decreased throughout the race, similarly to that which occurred in athletes at the Tor des Géants® (Tonacci et al., 2016), where a mix of strenuous physical effort and sleep restriction/deprivation negatively affected the olfactory function. Among the possible causes of the abovementioned sensory-cognitive detriment, sleep deprivation (or sleep restriction) was seen to be correlated with the “delta” of olfactory decrease between pre-race and post-race assessment. On the other hand, no particular effects for body composition variables were seen on cognitive domains, probably masked by other, predominant contributions, similarly to what reported during the Tor des Géants (Tonacci et al., 2016). This particular comparison was rarely performed among athletes, even though it was demonstrated that exercise-induced dehydration severely affects cognitive function (Cian et al., 2000). The findings reported by Cian et al. (2000) were referring, however, to dehydrated athletes, while none of our athletes had experienced dehydration as showed by the TBW composition.

Another cognitive domain, language fluency, was otherwise clearly improved during the race, with a similar—and opposite—dynamic when compared to olfaction. Interestingly, the cognitive domains studied with TMT—in particular those more related to the TMT-B score—and not different from each other between pre- and post-race assessment, were correlated with the COWAT score at the post-race assessment.

This different response detected with olfactory and cognitive testing is probably related to a differential activation of several cortical areas in response to stimuli. In fact, it could be stated that during the physical effort of the Transpyrénéa race, some cortical areas are more largely activated by the tasks required of the athlete in order to succeed in the competition, due to the so-called “neural efficiency” hypothesis (Vernon, 1993; Del Percio et al., 2009; Nakata et al., 2010).

This theory is based on psychometric considerations, and was strengthened by several neuroimaging studies using both ionizing radiation- (PET, SPECT) and non-ionizing radiation (fMRI)-based methods. Such research found that the subjects scoring highest on tests such as intelligence quotient, word fluency, spatial skills, and working memory show weaker fronto-parietal activation during cognitive tasks (Haier et al., 1988, 1992, 2004; Parks et al., 1988; Charlot et al., 1992; Rypma and D'Esposito, 1999; Rypma et al., 2002, 2005; Ruff et al., 2003).

Indeed, it is well-known that the cortical structures involved in olfactory identification processing (piriform and entorhinal cortex, amygdala, orbitofrontal cortex, anterior olfactory nucleus) are quite different when compared to the cortical structures called upon by COWAT for language fluency and execution (prefrontal cortex, caudate and subthalamic nuclei) and from the structures involved in tasks such as processing speed and attention (TMT-A) or visuomotor tracking, sequencing and cognitive flexibility (TMT-B).

During ultra-trail marathons or similar competitions, the athlete is called upon to process a number of stimuli that arise from the peculiarities of the race. It can be stated that ability in executing tasks, attention and visuomotor tracking is crucial for the good outcome of several sports activities (Abernethy, 1990; Ferrari and Rizzolatti, 2015), including walking, running or similar activities (Drew et al., 2008), in both good and bad visibility conditions (Durgin and Pelah, 1999). This could be the main reason why TMT and COWAT scores do not decrease throughout the race, and indeed are in some cases enhanced as seen with language fluency, whereas a cognitive pathway related to sensory stimuli, such as the olfactory path, could be somehow sacrificed for the advantage of more important cortical circuitries.

It can be hypothesized that this “double nature” of increasing the activity of some cortical parts while decreasing others that are less useful for the primary task to be accomplished (the competition in this case), could be related to some compensatory mechanisms of the brain, which occur in a wide number of disorders or sports injuries but to date have been reported in only a few studies on healthy athletes (Laurienti et al., 2002; Kudo et al., 2004). This could also be the reason why a correlation between cognitive parameters and sleep restriction was not found, contradicting some literature findings (Fullagar et al., 2015).

### Limitations

The main limitation of this study is the absence of information about weather conditions for each athlete. In fact, during a similar competition, held on a route of more than 800 kms, completely different weather conditions were encountered. At the beginning of the race at Le Perthus, the temperature was quite high (around 32 °C), whereas at T1 assessment most of the athletes arrived during a thunderstorm, at night and at around 9°C temperature. However, some of our athletes encountered rainy weather at high altitude (between T1 and T2), whereas other ones were resting during the storms. Therefore, it is nearly impossible to account for the weather conditions for all the athletes, and this could be considered a limitation of this study.

Furthermore, the subjects enrolled (*n* = 40) were a subgroup of participants (*n* = 242 starters) and not necessarily a “representative sample” of the overall athlete population, despite being age- and gender-matched with the overall population participating.

Finally, the bioelectrical impedance analysis for evaluating TBW could be considered in some instances to be a further methodological limitation of the current investigation.

### Conclusions

Summarizing, to the best of our knowledge, this is the first study investigating cognitive and neurosensory features in ultra-trailers performing a competition of more than 800 kms. Here, the bout of “psychocognitive stress” experienced by athletes during a strenuous competition such as the Transpyrénéa, where a massive physical effort is combined with sleep restriction (or for some periods, sleep deprivation), could transiently affect the overall cognitive balance of athletes. Since athletes are mainly required to enhance their attention, visuospatial processing and executive processing overall in order to safely conclude the competition, some other portions of the human cortex could be less stimulated, thus decreasing their activity. This process could occur in areas supervising olfactory processing, decreasing the ability of the subjects to correctly identify odors. According to previous evidence (see Tonacci et al., 2016 for an example), this deficit is probably temporary, with a normal olfactory function that would probably be restored in a few days or weeks, possibly depending on the athlete him/herself.

Future work is required to study similar domains on shorter trails, not massively impacted by sleep deprivation issues, and on competitions with a different altimetry profile, to evaluate the effect of high altitude, cold stress and in some instances hypoxia, on such variables. Furthermore, the cortical activity of the different brain areas mentioned in this work could be an interesting object of study for research involving ultra-trail marathons and similar competitions.

## Ethics statement

The study protocol was approved by the institutional Ethics Committee of the Aosta Valley Hospital (n. 895; 31/8/2015) and followed the guidelines of the Helsinki Declaration. All volunteers were informed regarding the design and purposes of the study and gave written informed consent.

## Author contributions

AT contributed substantially to the conception and design of the work, participated in data acquisition and analysis, as well as in the interpretation of results, drafted the work and approved its version to be submitted. SM contributed substantially to the conception and design of the work, participated in data acquisition and analysis, critically revised the work and approved its version to be submitted. KU contributed substantially to the design of the work, critically revised the work and approved its version to be submitted. FS contributed substantially to the conception of the work, critically revised the work and approved its version to be submitted. AF contributed substantially to the conception and design of the work, participated in data acquisition, critically revised the work and approved its version to be submitted. GG contributed substantially to the conception of the work, critically revised the work and approved its version to be submitted. RC contributed substantially to the conception of the work, critically revised the work and approved its version to be submitted. LP contributed substantially to the conception and design of the work, participated in data acquisition and analysis, as well as in the interpretation of results, drafted and critically revised the work, and approved its version to be submitted. All the authors agree to be accountable for all aspects of the work in ensuring that questions related to the accuracy or integrity of any part of the work are appropriately investigated and resolved.

### Conflict of interest statement

The authors declare that the research was conducted in the absence of any commercial or financial relationships that could be construed as a potential conflict of interest.

## Abbreviations

BMI: Body Mass Index
COWAT: Controlled Oral Word Association Test
CPs: Checkpoints
LBs: Life bases
NTST: Normalized total sleep time
PET: Positron Emission Tomography
SD: Standard Deviation
SPECT: Single-Photon Emission Computed Tomography
SPSS: Statistical Package for Social Science
TBW: Total Body Water
TMT: Trail Making Test
TST: Total sleep time.
